# Effects of transcutaneous electrical phrenic nerve stimulation and transcutaneous electrical diaphragmatic stimulation: A protocol for a randomized, single-blind, crossover trial

**DOI:** 10.1371/journal.pone.0347493

**Published:** 2026-04-27

**Authors:** Caroline Gomes Mól, Ricardo Kenji Nawa, Angélica Cristiane da Cruz Britto, Raquel Afonso Caserta Eid, Elaine Cristina de Campos, Leandro Teixeira Saraiva, Renato Fraga Righetti, Wellington Pereira Yamaguti

**Affiliations:** 1 Department of Critical Care Medicine, Hospital Israelita Albert Einstein, São Paulo, SP, Brazil; 2 Faculdade Israelita de Ciências da Saúde Albert Einstein, Hospital Israelita Albert Einstein, São Paulo, SP, Brazil; 3 Department of Medical and Surgical Clinical Care, Hospital Israelita Albert Einstein, São Paulo, SP, Brazil; 4 Rehabilitation Service, Hospital Sírio-Libanês, São Paulo, SP, Brazil; University of Kentucky, UNITED STATES OF AMERICA

## Abstract

**Background:**

Patients in the intensive care unit (ICU) are at high risk of developing respiratory muscle weakness, which may lead to prolonged mechanical ventilation and worsening clinical outcomes. Noninvasive neuromuscular electrical stimulation techniques, such as transcutaneous electrical phrenic nerve stimulation (TEPNS) and transcutaneous diaphragmatic electrical stimulation (TDES), offer potential therapeutic options for patients unable to generate voluntary active contractions. However, there is limited research directly comparing the effects of these modalities on diaphragmatic function. The primary objective of this study is to evaluate the effects of TEPNS and TDES on diaphragmatic function in healthy adults. Secondary objectives include assessing the safety, feasibility, and perceived discomfort associated with the application of each technique.

**Methods:**

This study will be a bicentric, randomized, single-blind, crossover trial that will be conducted at two hospitals in São Paulo, Brazil. Healthy adults aged 18–60, with a BMI between 18.5 and 24.9 kg/m^2^, no history of respiratory diseases, and no contraindications for the proposed electrical stimulation modalities will be included. Diaphragm thickness, thickening fraction, and mobility will be assessed using ultrasound during tidal breathing. Participants will be randomly assigned to begin the intervention with one of two experimental conditions: (1) TEPNS protocol or (2) TDES protocol. In addition, data on the safety and feasibility of each electrical stimulation modalities will also be collected.

**Trial registration:**

This protocol is registered at ClinicalTrials.gov under the number NCT06339632.

## Introduction

Critically ill patients are at high risk of developing both respiratory and limb muscle weakness [1]. Intensive care unit (ICU)-acquired muscle weakness is a well-established and common condition for limb muscles, strongly associated with poor clinical outcomes [2,3]. In contrast, the impact of respiratory muscle weakness has been less extensively studied; however, emerging evidence suggests it may contribute to prolonged mechanical ventilation (MV) support [4], an increased risk of ICU readmission [5], and higher mortality rates [6]. Diaphragm dysfunction affects more than 60% of patients undergoing MV support [7] and is associated with difficult and prolonged weaning from MV, as well as unfavorable clinical outcomes [4].

Over the past few years, several techniques have been proposed and developed to address respiratory muscle weakness, including invasive methods designed to induce diaphragm muscle contractions in patients with respiratory failure or muscle weakness [8,9]. These techniques, classified as transvenous approaches, involve the stimulation of the phrenic nerve—which controls the diaphragm movement—through electrodes surgically inserted into the internal jugular or subclavian [8–10]. This allows for direct activation of the diaphragm. However, invasive phrenic nerve stimulation methods present several drawbacks that limit their use in clinical practice. These include the risks of complications and adverse events such as hematoma, pneumothorax, thrombosis, and infection at the catheter insertion site [11].

Neuromuscular electrical stimulation (NMES) is a non-volitional and noninvasive therapeutic approach developed to prevent and treat muscle weakness. This technique involves the application of electrical stimuli through surface electrodes to induce effective muscle contractions [12,13]. Because NMES does not require active patient participation from the patient to elicit muscle contraction, it can be used even in clinical situations where the patient is under sedation and analgesia [12]. In this context, transcutaneous electrical phrenic nerve stimulation (TEPNS) and transcutaneous diaphragmatic electrical stimulation (TDES) have emerged as noninvasive modalities to induce contractions in diaphragm muscle fibers [14], offering a safer alternative to invasive techniques and procedures.

Noninvasive stimulation of diaphragm contraction has been investigated, however, the heterogeneity of protocols and NMES parameters used—combined with the anatomical complexity of locating the diaphragm’s motor point—limits the reproducibility and clinical applicability of this technique [15–17]. Therefore, the primary objective of this study is to compare the effects of TEPNS and TDES on diaphragm thickness, thickening fraction, and diaphragmatic mobility in healthy adults.

## Methods

### Study design

**Fig 1:**
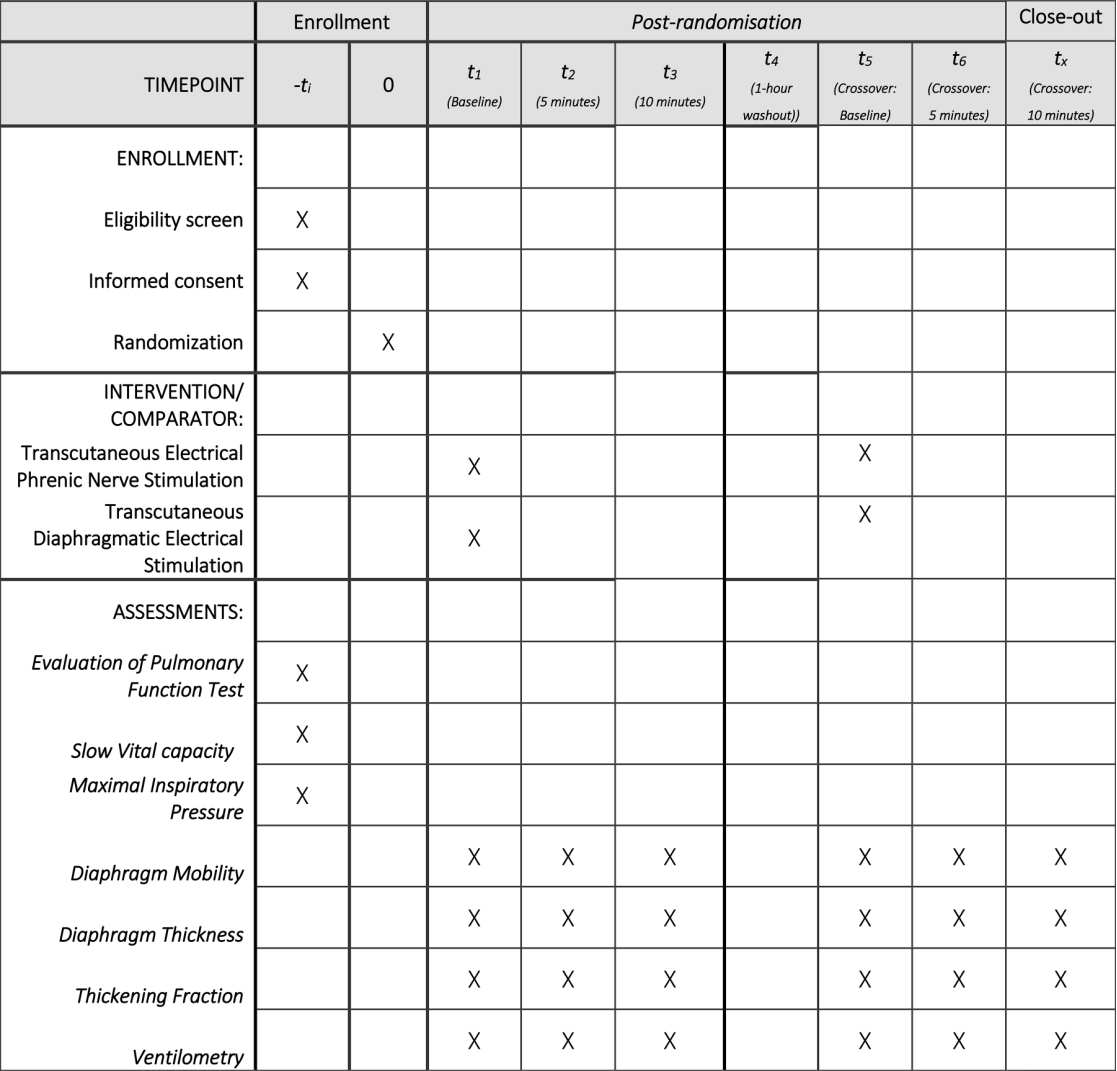
SPIRIT diagram.

**Fig 2:**
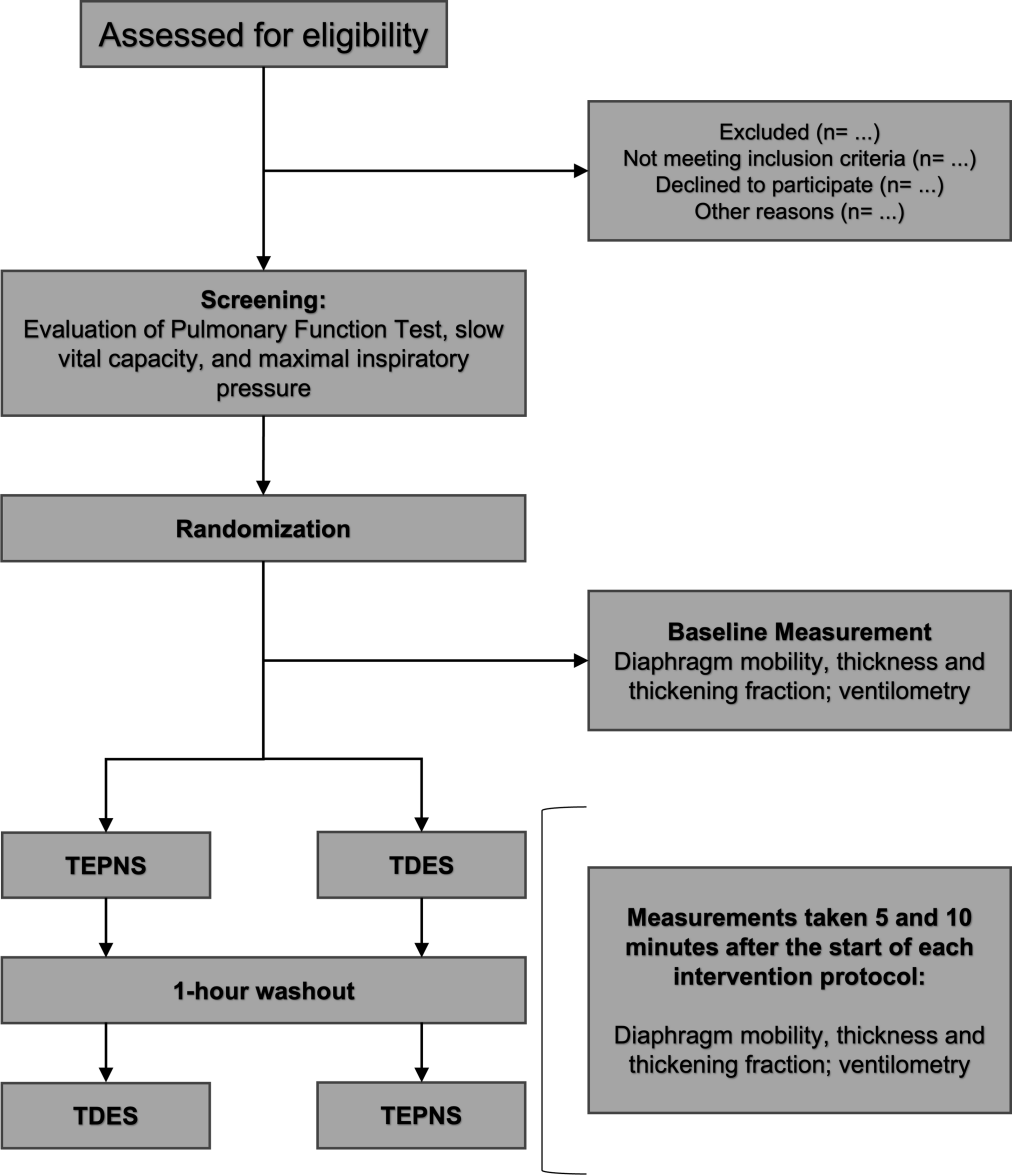
Flow diagram of the crossover trial. TEPNS: transcutaneous electrical phrenic nerve stimulation; TDES: transcutaneous diaphragmatic electrical stimulation.

This study will be a bicentric, randomized, single-blind, crossover trial, approved by the local Ethics Committee of the Hospital Sírio-Libanês (approval number 6.631.794, approved on February 1, 2024). The study is currently in the participant recruitment phase, which began on April 1, 2024, and has not yet generated any results, with recruitment expected to be completed by July 2027. The study protocol was prospectively registered in the *ClinicalTrials.gov* database (NCT06339632) and structured in accordance with the *Standard Protocol Items: Recommendations for Interventional Trials 2025* —SPIRIT 2025 guidelines (S1 File). The overall schedule and time commitment for trial participants are depicted in **Fig 1** and the study design is illustrated in **Fig 2**.

Participants will be recruited from the city and metropolitan region of São Paulo through announcements on corporate and institutional communication printed and electronic media distributed across the hospital units and educational facilities of Hospital Sírio-Libanês and Hospital Israelita Albert Einstein. Recruitment and initial contact will be conducted remotely. Prospective participants will be instructed to complete an initial application form, accessible via a link to the Research Electronic Digital Capture (REDCap) system. This form will provide an overview of the study’s scope and objectives, followed by a request for registration information. Eligible individuals will be contacted by the research team to provide written informed consent. Upon signing the written informed consent form, participants will be scheduled for a screening visit.

Inclusion criteria will require participants to be between 18 and 60 years of age, have normal pulmonary function as assessed by spirometry, and a body mass index (BMI) between 18.5 and 24.9 kg/m^2^. Exclusion criteria include current or former smokers; individuals with known cardiorespiratory diseases such as restrictive pulmonary diseases, chronic obstructive pulmonary disease (COPD), asthma, cystic fibrosis, or related conditions; individuals with implanted electrical devices, including pacemakers or implantable cardioverter-defibrillators; and individuals with contraindications to transcutaneous electrical stimulation or diaphragmatic assessment. Contraindications may include altered sensitivity, wounds at the application site, anatomical deformities, or other medical conditions.

Eligible participants will undergo a screening process to confirm compliance with the inclusion criteria. This includes a demographic interview, pulmonary function test using a calibrated Koko^®^ spirometer (PFT System), assessment of maximum inspiratory pressure (MIP), forced vital capacity (FVC) and BMI measurement. Pulmonary function tests will be conducted in accordance with the guidelines of the American Thoracic Society and the European Respiratory Society [18]. Participants exhibiting abnormal pulmonary function indicative of obstructive or restrictive disease, or a BMI outside the established range, will be excluded from the study.

### Sample size

Since no previous studies have evaluated the effects of this specific intervention on the diaphragm thickness, an initial pilot phase including 12 participants will be conducted to obtain preliminary estimates of variability and treatment effect. These data will be used to inform a formal sample size calculation for the main crossover trial. The primary outcome for sample size determination will be diaphragm thickness. The sample size calculation will be based on the expected within-participant mean difference between intervention conditions and the corresponding standard deviation derived from the pilot data, assuming a paired t-test framework. The required sample size will be estimated considering a two-sided significance level of 5% (α = 0.05) and a statistical power of 80%. Additionally, a potential dropout rate of approximately 20% will be incorporated to ensure adequate statistical power for the final analysis.

### Randomization and allocation

To minimize the potential influence of age on the results, initial allocation in this crossover study will be randomized in a balanced manner according to participants’ age, using three predefined age groups: (i) 18–35 years, (ii) 36–46 years, and (iii) 47–60 years, according to screening database. Participants will be randomly assigned to begin the intervention with either the TEPNS or TDES protocol. Randomization will be performed using a sealed-envelope method with sequentially numbered, opaque envelopes prepared according to a block randomisation scheme (block size = 4). Envelopes will be opened immediately prior to the baseline ultrasonography assessment. This procedure ensures balanced allocation and an equal probability (1:1) of initiating the intervention with either the TPNS or the TDES protocol.

### Blinding

Blinding participants to the TEPNS or TDES intervention is not feasible, as most individuals can perceive the electrical stimulation, and visible muscle contractions are required to confirm proper electrode placement. However, ultrasound images will be analyzed by a researcher who is blinded to the intervention procedure and image acquisition. Additionally, all file names will be coded and will not indicate the group to which the participant was allocated.

### Intervention

The intervention will be conducted in two different times, depending on the randomization sequence, and may begin with either TEPNS or TDES. A 1-hour washout period will be conducted between the two interventions. During interventions, participants will be instructed to breathe normally at tidal volume, using the perception of the electrical current as a cue for the onset of inspiration. This ensures synchrony with the stimulation cycle (“on” and “off” times), without altering the participant’s natural respiratory rhythm or pattern. For both the TEPNS and TDES, electrical stimulation will be applied continuously for 10 minutes in a single session.

### Transcutaneous electrical phrenic nerve stimulation (TEPNS)

**Fig 3:**
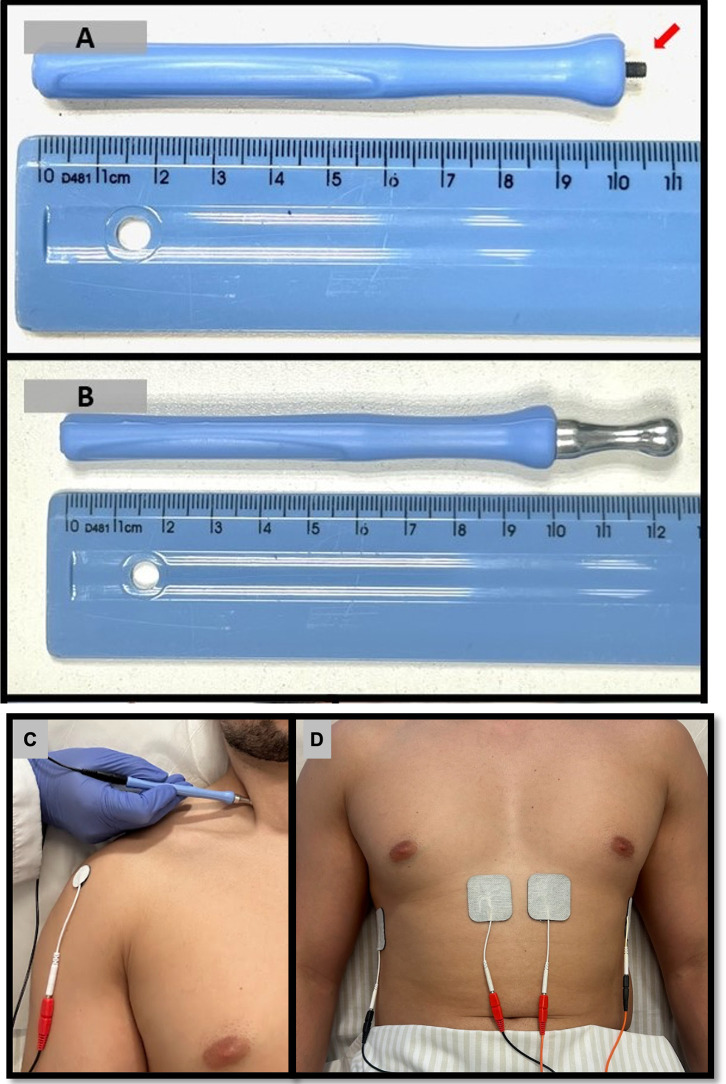
Electrostimulation technique: (A) 2 mm microcurrent stick, (B) microcurrent stick with connected spherical tip, (C) transcutaneous electrical phrenic nerve stimulation (TEPNS), and (D) transcutaneous diaphragmatic electrical stimulation (TDES).

The TEPNS parameters will be biphasic waves with a frequency of 10 Hz, a pulse width 200 μs, a rise time of 1.0 second, “on” and “fall” times of 1.0 second each, and a 2-second “off” time, resulting in 12 stimuli per minute [19]. TEPNS application will begin by identifying the right cervical phrenic nerve pathway using ultrasound, guided by anatomical landmarks including the anterior scalene muscle, sternocleidomastoid, internal jugular vein, and brachial plexus roots. The phrenic nerve appears as a hypoechoic (darker on ultrasound image), oval structure superficial to the anterior scalene muscle [20]. The active electrode (negative pole) will be placed along the nerve pathway between the heads of the sternocleidomastoid muscle using a 2 mm spherical microcurrent electrode (Neurodyn Esthetic, IBRAMED) (**Fig 3A and 3B**). The passive electrode (positive pole) will be positioned at the acromion shoulder to complete the electrical circuit [21] (**Fig 3C**). Stimulation intensity will be adjusted to the maximum level tolerated by the participant, sufficient to produce a diaphragmatic contraction confirmed by the ultrasound assessment without inducing contraction of the cervical or shoulder muscles [21].

### Transcutaneous diaphragmatic electrical stimulation (TDES)

The electrical parameters for TDES consist of biphasic waveform with a stimulation frequency of 30 Hz, a pulse width of 400 μs, a rise time of 1.0 second, an “on” time of 1.0 second, a fall time of 1.0 second, and an “off” time of 2 seconds, resulting in 12 stimuli per minute [19]. For TDES application, two self-adhesive electrodes (5.0 x 5.0 cm) will be placed bilaterally in the parasternal region, positioned inferior and lateral to the xiphoid process. An additional pair of electrodes will be placed bilaterally in the intercostal space between the 6th and 7th ribs, along the midaxillary line (**Fig 3D**). Electrode positioning will be guided by an initial ultrasound evaluation to identify the region of greatest visible diaphragmatic apposition. The stimulation intensity, measured in milliamperes (mA), will be gradually increased to the maximum level tolerated by the participant, until visible diaphragmatic contraction is achieved without concomitant contraction of abdominal muscles.

### Outcome measures

#### Diaphragmatic ultrasonography.

Diaphragmatic assessments will be performed using a LOGIQ E^®^ (R7) ultrasound system equipped with linear (GE12L-RS, 13 MHz) or a convex transducer (C1-5-D), in conjunction with water-based gel. Participants will be evaluated in supine position with a 30 degrees trunk inclination. Diaphragmatic mobility, thickness, and thickening fraction will be assessed. To measure tidal volume during the intervention, a facial mask will be connected to a ventilometer and a HEPA-filtered. The facial mask will be placed by the evaluator one minute before the assessment, and during two specific intervals: between the 4th and 5th and between the 9th and 10th minute of the intervention. Ultrasound evaluations will be conducted during spontaneous ventilation at tidal volume during both intervals, according to the randomization protocol.

Measurements of diaphragm mobility, thickness, and thickening fraction will be averaged from multiple respiratory cycles within the following three time points: (i) baseline (pre-intervention), (ii) 4–5 minutes into the intervention, and (iii) 9–10 minutes post-intervention. All ultrasound images will be exported to a secure storage device designated for research purposes only, with access restricted to authorized study personnel and researchers. While blinding is not feasible during image acquisition, a blind evaluator–who is not involved in the intervention or image collection–will perform the image analysis.

**Fig 4:**
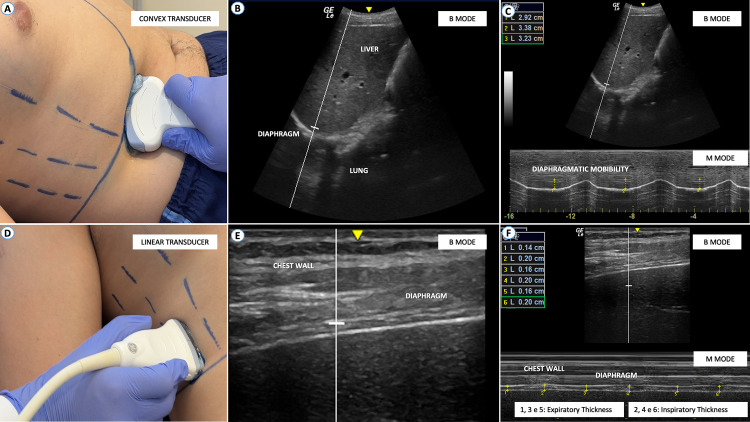
Technique for ultrasound evaluation of diaphragmatic muscle thickness: **A)** Convex transducer. **B)** Cake-shaped image on the screen in “brightness – B” mode. **C)** Mobility assessment in “motion – M” mode. Convex transducers are primarily used for abdominal exams due to their wider and deeper view. **D)** Linear transducer. **E)** Cake-shaped image on the screen in “brightness – B” mode. **F)** Mobility assessment in “motion – M” mode.

Diaphragmatic mobility will be assessed using a convex transducer (2–6 MHz) positioned in the right subcostal region along the mid-clavicular line, angled cephalically (**Fig 4A-4C**). Initially, B-mode ultrasound will be used to locate the diaphragmatic hemi-cupola. Once identified, M-mode will be employed to measure respiratory excursions, using the inferior vena cava and gallbladder anatomical landmarks. Mobility will be quantified as the vertical distance between the baseline position during expiration and the point of maximal diaphragmatic displacement during inspiration [22,23].

Diaphragmatic thickness (Tdi) and thickening fraction will be assessed using a high-frequency linear transducer (7–13 MHz) placed over the diaphragm's zone of apposition (ZA) (**Fig 4D-4F**). This zone is located between the 8th and 9th intercostal spaces, approximately 0.5 to 2.0 cm below the costophrenic angle (**Fig 4F**) [24,25]. The diaphragm will be identified as a hypoechoic muscle layer bordered by hyperechoic pleural and peritoneal membranes [26]. “Tdi” will be measured as a distance between the pleural and peritoneal lines at the end of expiration (Tdi-exp) and at the end of inspiration (Tdi-insp). The diaphragmatic thickening fraction will be calculated as the percentage increase in thickness from inspiration to expiration, using the following formula: [F_Ei_ = (E_i_ – E_e_ / E_e_) x 100]. Where “FEi” is the inspiratory muscle thickening fraction; “Ei” is the muscle thickness at the end of inspiration; and “Ee” is the muscle thickness at the end of expiration [23].

#### Sensory discomfort evaluation.

Sensory discomfort will be assessed using a horizontal numeric discomfort rating scale. The scale will be presented in printed form on standard 10.0 cm paper, where “0” indicates no discomfort and “10” represents the worst imaginable discomfort. Participants will be asked to rate their perceived sensory discomfort for both interventions (i.e., TEPNS and TDES) at three time points: (i) Baseline – before the start of the intervention; (ii) Measure 5 – the first re-evaluation, conducted between the 4th and 5th minute of the intervention; and (iii) Measure 10 – the final re-evaluation, conducted during the last minute of the intervention [27].

### Safety assessment

Safety criteria will be evaluated by monitoring for adverse events during the intervention period. Criteria for adverse events include: mean arterial pressure < 65 mmHg, heart rate > 140 bpm or < 50 bpm, occurrence of arrhythmias, oxygen saturation < 92%, elevated body temperature (> 37.7°C), reported discomfort > 7 on the visual analog discomfort scale, and burns or skin injury at the electrode application site. Participants’ vital signs will be continuously monitored throughout the intervention and recorded at 2-minute intervals using a multiparameter monitor (General Electric CARESCAPE Monitor B650).

All adverse events will be recorded and classified according to the World Health Organization’s patient safety guidelines [28], which ranges from “No harm” (no symptoms detected or observed, no treatment required) to “Death” (event caused or significantly contributed to short-term mortality). Adverse events will be monitored only during the intervention, as no long-term side effects related to transcutaneous electrical stimulation have been reported in the literature. Any adverse events will be documented, classified, and reported to the institutional authorities responsible for ethical oversight and study approval.

### Statistical analysis

The Research Electronic Data Capture (REDCap) platform will be used for data entry and storage. Continuous variables will be reported as mean ± standard deviation (SD) for normally distributed data, or as median and interquartile range (IQR, 25th–75th percentile) for non-normally distributed data. Categorical variables will be presented as absolute numbers and percentages. Normality will be assessed using the Shapiro–Wilk test. For within-participant comparisons between TEPNS and TDES conditions, paired t-tests will be used for normally distributed data, and the Wilcoxon signed-rank test will be applied for non-parametric data. The primary analysis will be performed using a linear mixed-effects model appropriate for a two-period crossover design, including treatment, period, and sequence as fixed effects, and participant as a random effect, thereby accounting for within-subject correlation. Percentage changes in diaphragm thickness, thickening fraction, and mobility will also be calculated.

The frequency of adverse events will be systematically recorded and descriptively analyzed to characterize the safety profile of both interventions. Additionally, associations between diaphragm thickness, thickening fraction, diaphragmatic mobility, stimulation intensity, participant-reported discomfort, and the occurrence of adverse events will be explored using Pearson’s or Spearman’s correlation coefficients, as appropriate. Adjustment for multiple comparisons will be performed where applicable to control the risk of type I error. Missing data will be addressed using multiple imputation by chained equations under the missing-at-random assumption. Each imputed dataset will be analyzed separately, and the results will be pooled using Rubin’s rules to obtain valid statistical inferences.

All statistical analyses will be performed using the Statistical Package for the Social Sciences (SPSS) version 28.0.1 (SPSS Inc., Chicago, IL, USA). A two-sided p-value < 0.05 will be considered statistically significant. The statistician responsible for data analysis will be blinded to the allocation sequence and intervention timing.

### Ethics and dissemination

This study will be conducted in accordance with Brazilian ethical standards for research involving human subjects and has been approved by the local Coordinating Research Ethics Committee (CAAE: 76562223.2.1001.5461; oppinion number 6.631.794). The study will be conducted at two private hospitals in São Paulo, Brazil.

### Modification of the protocol

Any changes to the protocol—including changes to study objectives, design, patient population, sample size, procedures, or other significant administrative aspects—will require a formal submission and approval by the institution, which also serves as the clinical trial registry.

### Confidentiality

To ensure participant confidentiality, each subject will be assigned a unique identification number for use throughout the study. All personal data will be stored securely in locked cabinets with restricted access, accessible only to authorized research personnel.

### Dissemination

The results of this study will be submitted for publication in peer-reviewed, open-access scientific journals and presented at relevant national and international scientific conferences.

### Patient and public involvement

No patients or members of the public were involved in the design, conduct, reporting, or dissemination of this research.

## Discussion

To our knowledge, this will be one of the first trials to directly compare two noninvasive electrical stimulation techniques applied to the diaphragm, TEPNS and TDES. Additionally, the randomized, single-blind, crossover design enhances internal validity while the bicentric conduction increases methodology rigor. Another strength of the present study protocol is the use of ultrasound to assess diaphragm thickness, thickening fraction and mobility, which provides an objective measure of diaphragm muscle recruitment and function.

Previous clinical investigations by Medrinal et al. [29] and Keogh et al. [30] reported no intervention-related adverse events associated with the use of non-invasive stimulation techniques applied to the diaphragm, supporting the safety of these approaches. In addition, no previous investigations have evaluated key ultrasound-derived parameters of diaphragmatic function such as diaphragm mobility, muscle thickness and thickening fraction as primary outcomes following stimulation interventions. The absence of prior data on these mechanistic endpoints precluded reliable assumptions regarding the expected effect size and consequently made an accurate a priori sample size calculation unfeasible. Therefore, an initial pilot phase including 12 participants was planned to provide preliminary estimates of variability and treatment effect. These data will be used to inform the sample size calculation for the main confirmatory crossover study described in this protocol.

The 1-hour washout period between stimulation conditions was defined to minimize the risk of potential carry-over effects in this crossover design. This interval was based on previous physiological and clinical studies on transcutaneous diaphragmatic electrical stimulation, suggesting that its effects on respiratory muscle activation, ventilatory parameters, and hemodynamic responses are predominantly acute and transient, with values returning to the baseline shortly after stimulation cessation [31].

The selection of a 10-minute stimulation period for both TDES and TEPNS was based on two primary factors: the time required to achieve a steady-state physiological response and the prevention of diaphragmatic fatigue and sensory discomfort in a single-session protocol. Previous studies investigating the acute effects of transcutaneous electrical stimulation on respiratory muscles have successfully used protocols ranging from 10 to 30 minutes to observe immediate changes in ventilatory patterns and autonomic modulation [31]. A 10-minute window is sufficient to stabilize the respiratory rate and heart rate variability parameters, allowing for reliable data collection while minimizing the risk of the subject's adaptation to the stimulus. In addition, since the goal was to assess the acute activation and functional response of the diaphragm, longer sessions could potentially induce contractile fatigue, especially in a crossover design. According to Zambon et al. [32] and Goligher et al. [33], the diaphragm is highly sensitive to changes in workload. Therefore, a shorter and controlled stimulation duration (10 minutes) was selected to ensure that the observed effects are due to muscular recruitment rather than a compensatory response to fatigue.

The choice of unilateral stimulation for TEPNS and bilateral stimulation for TDES in the present study was based on previously described application techniques for each modality. Specifically, transcutaneous phrenic nerve stimulation is conventionally performed unilaterally due to the anatomical accessibility of the phrenic nerve in the cervical region and to allow more selective activation of a single hemidiaphragm [30], whereas transcutaneous diaphragmatic electrical stimulation is typically applied bilaterally to optimize current distribution across the thoracoabdominal region and to promote more global diaphragmatic recruitment [31,34]. Despite this methodological difference, diaphragmatic assessment in this study was standardized by evaluating a single hemidiaphragm, in accordance with prior ultrasound-based studies demonstrating that unilateral assessment provides reliable and reproducible estimates of diaphragmatic thickness, thickening fraction, and mobility [17,35]. This approach is widely adopted due to technical feasibility and a more consistent acoustic window, particularly on the right side, where the liver provides an optimal sonographic window that improves image quality and measurement reliability [17]. Therefore, although stimulation strategies differed between interventions, outcome measurements were standardized to ensure comparability between conditions and to reduce measurement variability.

Despite the strengths, some limitations should be acknowledged. The study will be conducted with a sample of health volunteers, which may limit the generalizability of the findings to critically ill patients. Healthy volunteers were defined as the initial study population to allow a controlled evaluation of feasibility, safety, and discomfort, prior to assessing the outcome of the intervention in critically ill patients. However, this approach may limit the external validity and generalizability of the findings. The nature and characteristics of the intervention was not designed to evaluate long term clinical outcomes. The findings of this study should be interpreted within the context of the specific sample, as they are limited to participants recruited from two hospitals in São Paulo, Brazil. Therefore, further studies with larger sample sizes and conducted across different geographic settings are warranted to confirm and expand upon these results, as well as to better evaluate potential differences between TEPNS and TDES. In addition, this study includes only adults (aged <60 years), which may limit the generalizability of the findings to older populations.

Furthermore, our study did not include a sham stimulation condition, as the primary objective was to compare the efficacy of two techniques already used in clinical practice for non-invasive diaphragmatic stimulation. Additionally, participant blinding was not feasible, since the stimulation methods (TEPNS and TDES) are applied to different anatomical locations, making it possible for participants to distinguish between the interventions.

Despite its limitations, this trial is expected to generate clinically relevant data, particularly regarding the safety of the interventions, which will be important to inform future studies in patient populations. The evidence generated by this trial will be especially important for advancing strategies aimed at mitigating respiratory muscle weakness.

## Supporting information

S1 File Standard Protocol Items: Recommendations for Interventional Trials 2025 (SPIRIT 2025). (PDF)
